# The Comparison of Patient Characteristics, Therapy Outcome, and Sexual Functions in Vaginismus Patients Prior to and During the COVID-19 Pandemic

**DOI:** 10.7759/cureus.52612

**Published:** 2024-01-20

**Authors:** Ebru E Zulfikaroglu

**Affiliations:** 1 Obstetrics and Gynecology, Eva Women's Health Clinic, Ankara, TUR

**Keywords:** griss, fsfi, female sexual dysfunction, vaginismus, covid-19

## Abstract

Background

The objective of this study conducted at one center is to compare the demographic features and female sexual functions of patients treated for vaginismus before the COVID-19 pandemic to those treated for vaginismus during the pandemic.

Aim

Additionally, the study intends to evaluate the results of vaginismus therapy and assess the post-treatment sexual functioning of women.

Methods

A retrospective analysis was conducted on the medical records of patients diagnosed with vaginismus who sought treatment between March 2018 and March 2022. The enrolled patients were categorized into two groups: the pre-COVID-19 group and the COVID-19 group. The following data have been collected: age, education level, occupation, and marriage duration. After three months of treatment, the patients were called for a follow-up examination and evaluation of their sexual functions.

Outcomes

Compared to the pre-COVID-19 group, an increase of 52.51% was observed in the number of patients admitted for treatment in the COVID-19 group.

Results

The severity of vaginismus in the patients was similar in both groups. There were no statistically significant changes observed in any of the areas of the Female Sexual Function Index (FSFI) scale.

Clinical implications

According to our findings, there was no significant difference in female sexual functioning between women who had vaginismus treatment during the pandemic and those who underwent pre-pandemic treatment.

Strengths and limitations

The research sample comprised women who sought medical care at our women's health clinic.

Conclusions

We believe that vaginismus patients who had previously avoided seeking treatment are now seeking it during the pandemic.

## Introduction

The COVID-19 pandemic, which emerged in China in December 2019, saw a fast global spread and was officially designated as a pandemic by the World Health Organization (WHO) on March 11, 2020 [1]. The virus has been widely recognized as a significant global health concern, leading to the implementation of various measures on a global scale aimed at mitigating its transmission. Given the importance of minimizing human-to-human interaction, these measures have significantly influenced several facets of everyday life, leading to adverse consequences on individuals' psychological welfare [2].

Vaginismus is categorized as a specific type of genito-pelvic pain/penetration disorder in the Diagnostic and Statistical Manual of Mental Disorders, Fifth Edition (DSM-5) [3]. This condition is characterized by recurring pain or the inability to engage in various forms of vaginal penetration, including tampon use, digital insertion, vaginal dilator application, gynecologic examinations, and sexual intercourse [4].

The COVID-19 pandemic caused a significant decline in sexual activity [5]. The global outbreak of COVID-19 has significantly transformed social dynamics worldwide, mostly due to the implementation of governmental restrictions and the pervasive fear of infection experienced by the general populace. The rapidity with which these alterations occurred left us feeling inundated, since there was no time for our mental and physical faculties to acclimate to the novel circumstances. The global state of affairs has elicited distress among individuals, leading to a profound examination of their emotional resilience in light of the persistent inundation of visuals depicting illness and mortality [5].

The research conducted on alterations in sexual behavior within the epidemic yielded inconclusive findings. Adults have reported a decline in the frequency of engaging in sexual activity and casual sex, as well as an increased likelihood of experiencing sexual dysfunction, engaging in masturbation, and using pornography [6]. Rogowska et al. [6] observed a decline in sexual pleasure during the pandemic in comparison to the year preceding it. In contrast to the pre-pandemic era, the frequency of sexual activity exhibited an increase during the period of lockdown [6]. Given the lack of definitive information about the impact of lockdown-related constraints on sexual activity, further investigation is warranted to elucidate the alterations in sexual behavior seen during the COVID-19 pandemic.

Several research have been conducted to examine the effects of the COVID-19 pandemic on female sexual function [7,8]. To the best of our understanding, there is a paucity of studies examining the impact of the COVID-19 pandemic on sexual function specifically for women diagnosed with vaginismus. The objective of this study conducted at one center is to examine and compare the clinical features of patients with vaginismus who had treatment before and during the pandemic. Additionally, the study intends to evaluate the results of vaginismus therapy and assess the post-treatment sexual functioning of women.

## Materials and methods

A retrospective analysis was conducted on the medical records of patients diagnosed with vaginismus who sought treatment at the women's health clinic in Ankara, Turkey, between March 2018 and March 2022. The enrolled patients were categorized into two groups: the pre-COVID-19 group and the COVID-19 group. The pre-COVID-19 era encompassed the time frame from March 2018 to March 2020, whereas the COVID-19 era extended from March 2020 to March 2022. The present investigation was carried out in adherence to the principles outlined in the Declaration of Helsinki. Consequently, prior to their inclusion in the study, all participants provided informed consent. The retrospective and observational nature of the research obviated the need for ethics committee approval. The inclusion criteria encompassed individuals who were diagnosed with vaginismus and were 18 years of age or older and who provided their agreement to participate in the study. The present study comprised a cohort of 351 participants who were selected based on the absence of specific exclusion criteria. These criteria encompassed the following conditions: (1) pregnancy at the time of the research, (2) the presence of vulvar vestibulitis syndrome, (3) a history of tubo-ovarian abscess and pelvic inflammatory disease, (4) the presence of sexually transmitted diseases, (5) reluctance to complete the questionnaires during a telephone interview, and/or (6) incomplete data. Each participant underwent individual interviews conducted by a qualified professional who specialized in both sex therapy and gynecology.

The following data have been collected: age, education level, occupation, and marriage duration.

The physician gathered the history of the couple and the patient, followed by doing a physical examination and a delicate gynecologic assessment to provide a definitive diagnosis while providing reassurance. The Lamont categorization system, which consists of four degrees of severity, was employed in our study through the utilization of gynecologic examinations. The utilization of gynecologic examination and categorization facilitated the identification of potential organic issues and the determination of appropriate therapeutic approaches. The assessment of vaginismus severity before therapy facilitated the provision of adequate assistance to these individuals during the treatment process. Therapeutic interventions were administered in accordance with the established protocols of the facility. Cognitive and behavioral therapies were applied to vaginismus patients as detailed in our previously published article [9].

After three months of treatment, the patients were called for a follow-up examination and evaluation of their sexual functions. Once more, the aforementioned experienced sex therapist and gynecologist carried out individual interviews with each participant at this juncture. The interviews were carried out with a standardized questionnaire that had been developed previous to the recruitment of participants, but its validation had not been established.

In this study, we utilized the Female Sexual Function Index (FSFI) scale [10], the female iteration of the Golombok-Rust Inventory of Sexual Satisfaction (GRISS) [11], and the Hamilton Depression Rating Scale (HDRS) [12].

The acquired data underwent statistical analysis using the Statistical Package for Social Sciences (SPSS) version 25.0 (IBM SPSS Statistics, Armonk, NY) for Windows (WIN). The analysis employed the following particular methods: (1) Descriptive statistics were employed to analyze the general and clinical characteristics of the subjects, (2) the clinical outcomes of the group were assessed using the chi-squared and independent t-tests, and (3) the chi-squared and multivariate logistic regression tests were utilized to examine disparities in the rate of complications between groups.

## Results

Demographic characteristics

A total of 351 patients in the research group had a mean age of 28.9 ± 5.2 (20-47) in the pre-COVID-19 group (n = 139) and 27.4 ± 4.9 (20-43) in the COVID-19 group (n = 212). The predominant level of education for both groups was a bachelor's degree, and most categorized themselves as "housewives." Most of the marriage durations were between 1.5 and five years. Compared to the pre-COVID-19 group, an increase of 52.51% was observed in the number of patients admitted for treatment in the COVID-19 group (Table 1).

**Table 1:** Demographic data of the study population Data are presented as n (%). Age data are presented as mean ± SD SD: standard deviation

Characteristics	Pre-COVID-19 group	COVID-19 group
Number	139	212
Age	28.9 ± 5.2	27.4 ± 4.9
Education level		
Illiterate	3 (2.15%)	0
Under diploma	12 (8.63%)	8 (3.77%)
Diploma	43 (30.93%)	52 (24.52%)
Bachelor's degree	67 (48.2%)	119 (56.13%)
Master of science	11 (7.91%)	22 (10.37%)
Doctorate	3 (2.15%)	11 (5.18%)
Occupation status		
Employee	14 (10.07%)	16 (7.54%)
Public servant	26 (18.7%)	45 (21.22%)
Business	12 (8.63%)	22 (10.37%)
Worker	13 (9.35%)	24 (11.32%)
Retired	1 (0.71%)	0
Housewives	73 (52.51%)	105 (49.52%)
Marriage duration		
1.5-5 years	97 (69.78%)	116 (54.71%)
6-10 years	27 (19.42%)	79 (37.26%)
>10 years	15 (10.79%)	17 (8.01%)

Treatment outcome

The severity of the vaginismus of the patients was similar in both groups (Table 2).

**Table 2:** Comparison of the severity of vaginismus, number of treatment sessions, and treatment outcome Patient's data are presented as n (%). Treatment session data are presented as mean (minimum-maximum) *Statistically significant difference (p ≤ 0.05)

Degree of vaginismus	Number of patients		Number of treatment sessions		Success rate	
	Pre-COVID-19	COVID-19	Pre-COVID-19	COVID-19	Pre-COVID-19	COVID-19
1st (mild)	44 (31.65%)	61 (28.77%)	2.1 (1-3)	2.2 (1-3)	100%	100%
2nd (moderate)	52 (37.41%)	82 (38.67%)	2.6 (2-3)	2.7 (2-3)	100%	100%
3rd (moderate-severe)	30 (21.58%)	55 (25.94%)	3.1 (3-4)	3.2 (3-4)	100%	100%
4th (severe)	13 (9.35%)	14 (6.61%)	4.2 (4-5)*	4.4 (4-5)*	100%	100%
Total	139	212	2.7 (2-5)	2.8 (2-5)	100%	100%

As seen in Table 2, in both groups, the number of treatment sessions in women with first-degree severity ranged from one to three; for minor severity, the number of treatment sessions ranged from two to three; the number of treatment sessions for third-degree severity ranged from three to four; for fourth-degree severity, the number of treatment sessions ranged from four to five. Following the intervention and a subsequent three-month period of observation, it was determined that all couples in both experimental groups achieved effective sexual intercourse (Table 2). The findings of this study indicate a notable association between the severity of vaginismus and the quantity of therapy sessions. At the time of the study, none of the participating couples had acquired the COVID-19 infection. Based on the input provided three months post hospital release, there were no reported instances of vaginismus recurrence.

Information about sexual function

Each woman had a routine gynecologic examination three months after their first sexual encounter, and the FSFI, GRISS, and HDRS were filled out by the women.

The participants' FSFI scores exhibited greater values prior to the onset of the pandemic, yet this observation did not provide statistically significant results. During the pandemic, a decrease in the domains of the FSFI, namely, desire, arousal, lubrication, and orgasm, was seen, although levels of satisfaction and pain remained unaffected. The study found that levels of satisfaction and pain remained consistent both prior to and after the epidemic. Nevertheless, the data analysis revealed no statistically significant difference, as indicated in Table 3.

**Table 3:** Results of the FSFI scores for the pre-COVID-19 and COVID-19 groups Data are presented as mean ± SD SD, standard deviation; FSFI, Female Sexual Function Index

Domain	Pre-COVID-19	COVID-19	p
Total score	21.7 ± 4.5	21.2 ± 4.1	0.28
Desire	3.6 ± 1.6	3.4 ± 1.3	0.21
Arousal	3.5 ± 1.6	3.2 ± 1.1	0.11
Lubrication	4.0 ± 1.5	3.6 ± 1.6	0.19
Orgasm	3.2 ± 1.1	3.1 ± 1.1	0.48
Satisfaction	3.9 ± 1.6	3.9 ± 1.5	0.61
Pain	3.5 ± 1.4	3.5 ± 1.3	0.98

The pre-COVID-19 group exhibited GRISS total scores of 28.2 ± 9.9 (ranging from 15 to 49), while the COVID-19 group had scores of 23.4 ± 14.2 (ranging from 9 to 55). The overall score of the participants and the specific categories of infrequency, non-communication, avoidance, vaginismus, and non-sensuality showed considerable improvement. Nevertheless, there was no statistically significant impact on the domain of dissatisfaction, but there was a notable decrease in scores related to anorgasmia (Table 4).

**Table 4:** Results of the GRISS scores for the pre-COVID-19 and COVID-19 groups Data are presented as mean ± SD *Statistically significant difference (p ≤ 0.05) SD, standard deviation; GRISS, Golombok-Rust Inventory of Sexual Satisfaction

Domain	Pre-COVID-19	COVID-19	p
Infrequency	4.3 ± 1.4	3.5 ± 2.3	0.008*
Non-communication	4.2 ± 1.8	3.0 ± 1.6	0.003*
Non-sensuality	4.4 ± 1.5	2.6 ± 1.3	0.004*
Avoidance	3.9 ± 1.4	1.5 ± 1.1	0.008*
Dissatisfaction	3.9 ± 2.4	4.1 ± 2.4	0.48
Vaginismus	4.1 ± 2.1	2.4 ± 1.1	0.003*
Anorgasmia	3.7 ± 1.4	5.1 ± 2.1	0.004*
Total score	28.2 ± 9.9	23.4 ± 14.2	0.004*

In the pre-COVID-19 group, the average HDRS score was 9.17 with a standard deviation of 5.21, ranging from 2 to 20. In contrast, the COVID-19 group had an average HDRS score of 14.89 with a standard deviation of 6.23, ranging from 6 to 27. During the COVID-19 pandemic, there was a notable decrease in mental well-being, suggesting a moderate depressive condition that was not associated with vaginismus (Table 5).

**Table 5:** Results of the HDRS scores for the pre-COVID-19 and COVID-19 groups Data are presented as mean ± SD *Statistically significant difference (p ≤ 0.05) SD, standard deviation; HDRS, Hamilton Depression Rating Scale

	Pre-COVID-19	COVID-19	p
HDRS	9.17 ± 5.21	14.89 ± 6.23	0.001*

## Discussion

The COVID-19 pandemic has developed as a serious public health catastrophe affecting all parts of society. Many areas of everyday life have been altered radically as a result of the social isolation caused by the pandemic's closures and limitations. This has had a profound influence on our spiritual life, including our sexual health, which we sometimes neglect. Given the research on the growth in marital and psychosexual disorders during the pandemic [13,14], it is critical to understand the patient profile of those who sought vaginismus therapy during the pandemic. There has been no previous research that compares vaginismus therapy and patient characteristics during the current pandemic to a comparable time period prior to the outbreak. The purpose of this research was to compare the demographic features and female sexual functions of patients treated for vaginismus before the COVID-19 pandemic to those treated for vaginismus during the pandemic.

We mentioned that we classified our patients into two groups: pre-COVID-19 and COVID-19. The pre-COVID-19 era was from March 2018 to March 2020, while the COVID-19 term was from March 2020 to March 2022. When we analyzed the demographic features of the two groups, we noticed some intriguing results. The number of patients admitted for treatment increased by 52.51% in the COVID-19 group compared to the pre-COVID-19 group. The mean ages of women with vaginismus in the pre-COVID-19 and COVID-19 groups in this research were 28.9 and 27.4 years, respectively. Although not statistically significant, we found a little drop in the average age of the patients who applied for therapy. The COVID-19 group had a higher education level than the pre-COVID-19 group. There was an increase in the number of COVID-19 patients applying for treatment in each of the bachelor's, master's, and doctoral degree subgroups. When analyzing the occupation status of the groups, it was observed that the COVID-19 group experienced a decrease in the number of patients identifying themselves as employees and housewives. Conversely, there was a proportional increase in the subgroups of public servants, business professionals, and workers who have a profession within the COVID-19 group. The duration between marriage and admission to the clinic for treatment was found to be the most frequent (1-5 years) in both groups in this research, which is consistent with the literature. According to several research, the average length of marriage before contacting a physician is 15-26 months [15,16]. The intriguing result for the marital duration data is that the COVID-19 group had a substantial rise in the 6-10-year subgroup.

There have obviously been many changes in our lives as a result of the COVID-19 pandemic restrictions, but the most basic result has been that couples have had to spend more time with each other. This, we believe, is reflected in our patients who have applied for vaginismus treatment. We first notice that more people seek therapy when we compare the demographic characteristics of the patients in our two study groups and assess the results holistically. Furthermore, more educated and professional individuals who postponed therapy prior to the pandemic sought vaginismus treatment.

Various therapy strategies have been established for the management of vaginismus, encompassing a range of approaches including psychoanalysis and surgical procedures [17]. In contemporary practice, a growing preference has emerged for the utilization of combination treatments in the treatment of vaginismus. These therapeutic approaches often involve the implementation of progressive muscle relaxation techniques, vaginal dilation exercises, and psychological counseling interventions. The categorization of individuals diagnosed with vaginismus based on the extent of symptom severity might potentially aid in identifying the most effective therapeutic strategies for patients exhibiting heightened degrees of discomfort, fear, and anxiety. Cognitive and behavioral therapy (CBT) has been identified as a significant intervention in the field of sexual functioning, with research demonstrating its efficacy in several areas. Significant research findings have demonstrated that CBT has the potential to yield positive outcomes in several aspects of sexual behavior, including increased frequency of sexual intercourse, enhanced communication on sexuality, heightened sexual pleasure, reduced avoidance behavior, and improved sensuality [18]. All patients with first-, second-, and third-degree severity received CBT, as we detailed in our previous article [9]. All patients with fourth-degree severity received it following medical hypnosis. This combined vaginismus treatment technique consists of CBT, expansion exercises, hypnotherapy (if needed), and post-procedure counseling and support. In our study, we observed that the degree of vaginismus, the average number of treatment sessions, and the success rate were commensurate between the two groups. The findings of our study indicate a statistically significant association between the severity of vaginismus and the frequency of therapy sessions in both groups. The findings presented in this study are corroborated by other scholarly works, as evidenced by the works of Eserdag et al. [17], Jeng et al. [19], and Pacik and Geletta [20]. All patients within both experimental groups exhibited effective sexual intercourse after therapy and over the three-month follow-up period. The patients did not disclose any instances of recurrence as feedback.

The research findings about the effects of the pandemic on female sexual function display contradictions. A limited number of comprehensive research have examined the collective sexual functioning of women during the pandemic, with the majority of these studies using the FSFI scale as a measurement tool. Multiple studies have demonstrated a decline in women's general sexual function, albeit with limited examination of sexual distress levels, which are essential for defining sexual dysfunction [21-23]. In a study conducted by Fuchs et al. [21] in Poland, a total of 746 women were examined. The researchers observed that the average total score on the FSFI was 30.1 prior to the occurrence of the pandemic. However, following the pandemic, this score decreased to 25.8. Concurrently, there was an increase in the percentage of women exhibiting dysfunction, with the proportion rising from 15.3% to 34.3%. The documented factors contributing to diminished sexual functioning encompassed factors such as interpersonal detachment from one's partner, less sexual desire stemming from heightened stress levels, interpersonal disagreements with one's partner, and apprehension around the transmission of the virus. Ilgen et al. [22] conducted a study wherein they observed no statistically significant differences in the overall FSFI score when comparing levels before and during the pandemic. There was a significant correlation observed between a decline in the sexual function score and an elevation in anxiety levels. Numerous studies have documented a general decrease in sexual function within the COVID-19 pandemic [23]. Moreover, evidence suggests that women have been more heavily impacted by this decline in sexual function compared to men [23].

During the pandemic period, females exhibited significantly lower total FSFI scores compared to the prior time. This decrease in scores might be attributed to the higher prevalence of anxiety and depressive symptoms, as well as increased stress perception, among females in comparison to males. A decline was noted across all areas of the FSFI score; however, statistical analysis did not reveal any significant differences in the domains of sexual desire, arousal, and pain. This phenomenon can be attributed to a rise in emotional intimacy and bonding, as well as beneficial transformations in the spousal or partner relationship, as reported by over 30% of individuals of both genders amidst the pandemic [24].

The group of women who did not engage in employment activities throughout the pandemic exhibited the most significant decline in their FSFI score in comparison to their counterparts who worked remotely from home [21]. The individuals who were employed in non-domestic settings exhibited the least significant disparity. This observation suggests that within the cohort of women who are not engaged in employment, the factors contributing to the decline in FSFI scores may be attributed to a dearth of physical activity and the exhaustion resulting from their daily routines. The study conducted by Lee et al. [25] provides evidence of the influence of family income on sexual life satisfaction. Specifically, the findings suggest that women who engage in regular employment experience reduced stress levels and enjoy the benefits of a consistent wage, perhaps resulting in a higher degree of sexual happiness compared to women who do not participate in the workforce [21].

On the other hand, there is very little literature on the female sexual functions of women with vaginismus during the pandemic [4,26]. One of these research reports findings from re-evaluating the FSFI scores of individuals previously treated for vaginismus during the pandemic period [26]. The study demonstrated a deterioration in depression scores throughout the pandemic, although the frequency of sexual intercourse remained unaffected. Additionally, improvements were observed in pain scores and total FSFI scores. Nevertheless, it was observed that the FSFI satisfaction and lubrication subscales exhibited somewhat smaller improvements in comparison to the other domains [26].

Our present study is a unique investigation of the correlation between female sexual function and the COVID-19 pandemic among women who had treatment for vaginismus before and during the COVID-19 pandemic. Although our analysis revealed that the FSFI values were comparatively lower among women who received treatment for vaginismus, the difference observed did not reach statistical significance. Furthermore, there were no statistically significant changes observed in any of the areas of the FSFI, including desire, arousal, lubrication, orgasm, satisfaction, and pain, when comparing pre-pandemic and post-pandemic periods.

Throughout the course of the pandemic, there has been an observed increase in the overall score of the GRISS scale, accompanied by a reduction in the domains associated with anorgasmia [4]. Our data indicate that women exhibited higher HDRS scores during the pandemic, potentially attributable to the psychological consequences of the global health crisis. These repercussions encompass concerns over viral transmission, anxiety stemming from social isolation, and the impact of lockdown measures. According to the GRISS anorgasmia subscale, there was a decline in the quality of sexual intercourse during the pandemic, despite the consistent frequency of sexual activity and a drop in reported pain levels as measured by the visual analogue scale. The apprehension of acquiring a disease from a sexual partner through activities such as kissing or other forms of intimate physical contact has the potential to hinder sexual performance. The potential presence of phobic reactions among the individuals under study may have influenced their experience of depressive and anxious symptoms in relation to the ongoing pandemic [27]. The pandemic has given rise to a set of enduring psychological manifestations, including worry, concern, and discomfort, which have been designated as "coronaphobia." These symptoms are shown to be more prevalent among those who exhibit anxiety tendencies and possess a background of mental health issues [28]. The correlation between anxiety and melancholy and their impact on pain severity and sexual issues in women with vaginismus has been shown, indicating that a circumstance characterized by high levels of stress, such as a lockdown, may lead to a decline in sexual function [28]. The observed increase in the GRISS anorgasmia subscale during the pandemic might likely be linked to the observed changes in interpersonal dynamics among couples, as shown by our research findings.

Despite the pandemic, none of the women in our research experienced recurring vaginismus, and they continued to enjoy pleasurable penetration with a drop in pain ratings; this might explain why GRISS scores improved despite the worldwide outbreak.

There exist some restrictions pertaining to this study endeavor. The research sample comprised women who sought medical care at our women's health clinic. However, it is important to note that our study is constrained by the incomplete consent obtained from all women receiving treatment for vaginismus. This might cause a bias in the demographics of our patient group when compared to its true state.

## Conclusions

In conclusion, vaginismus, which is well recognized as a prevalent sexual disorder, has a substantial influence on the quality of intimate partnerships. Our study findings indicate that there was no significant alteration in female sexual functions among women undergoing treatment for vaginismus throughout the pandemic. Despite the heightened levels of stress and sadness experienced, a majority of the participants demonstrated improvements in various aspects of sexual function. The COVID-19 epidemic has imposed several limits that have significantly impacted our lives. One notable consequence of these constraints is the increased amount of time couples have had to spend together. Due to this rationale, it is hypothesized that individuals afflicted with vaginismus, who had previously deferred seeking treatment, subsequently pursued therapeutic interventions in the wake of the pandemic. Furthermore, throughout the COVID-19 era, we noticed that more educated and professional patients sought treatment. The integration of cognitive and behavioral therapy, dilatation exercises, post-procedure counseling, and psychological support demonstrates a promising and secure method for addressing vaginismus, with positive outcomes.
